# Supplementary material for: Ocular Symptoms Associated with COVID-19 Are Correlated with the Expression Profile of Mouse SARS-CoV-2 Binding Sites
Source: Viruses. 2023 Jan 26;15(2):354. doi: 10.3390/v15020354 (PMC9961464; doi:10.3390/v15020354)
Supplement: Supplementary file 1 [file viruses-15-00354-s001.zip › viruses-2131649-supplementary.pdf]

Supplementary Files for

Ocular Symptoms Associated with COVID-19 Are Correlated with the Expression Profile of Mouse SARS-CoV-2 Binding Sites

Julien Brechbühl, Flavio Ferreira, Ana Catarina Lopes, Emilly Corset, Noah Gilliand and Marie-Christine Broillet

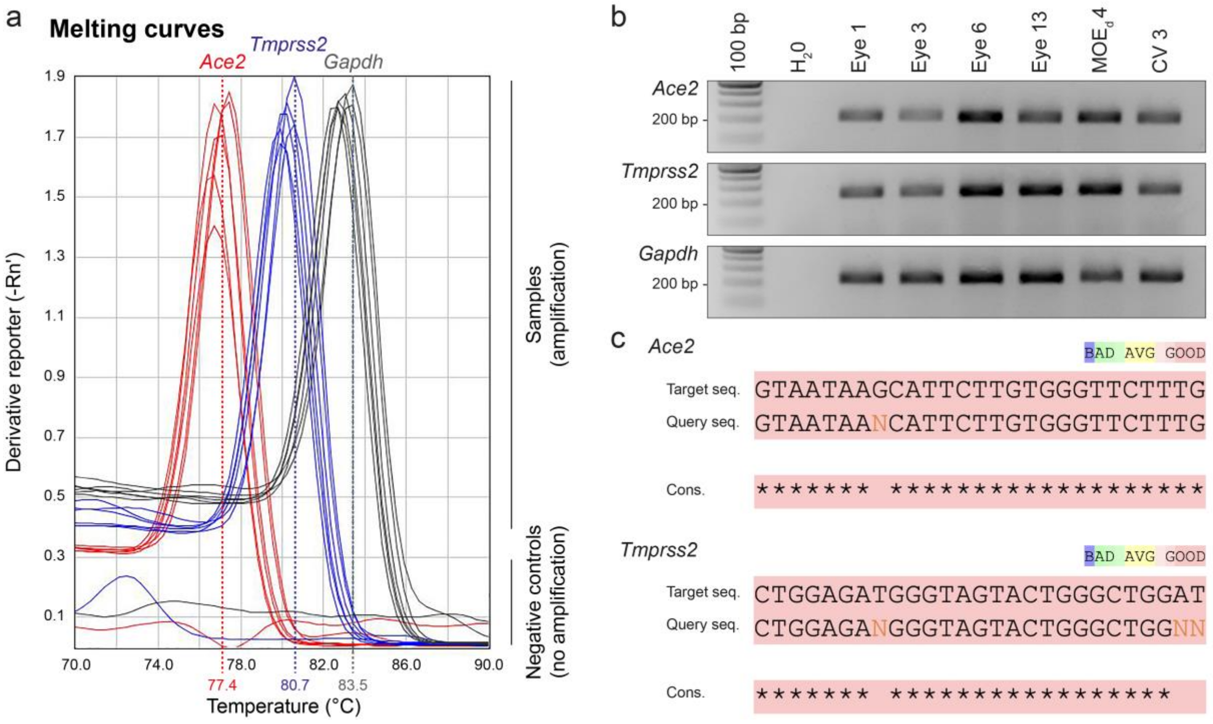

Figure S1. Validation of the fragment amplifications specificity. (**a**) Representative RT-qPCR melting curve analysis revealing pure and unique amplicons. The derivative reporter of the normalized fluorescence (-Rn′) method was used to identify a single melting temperature peak (Tm) for each investigated gene (*Ace2*, in red; *Tmprss2*, in blue; *Gapdh*, in black). Here, samples used were from eyes, dorsal MOE and CV tissues (Eye1, Eye3, Eye6, Aye13, MOE$_d$4 and CV3) obtained from mice ($Gfp^{-/-}$). No amplification was observed in negative controls (H_2O). (**b**) Electrophorese analysis of RT-qPCR amplifications highlighted single fragment products. (**c**) After sequencing processes, local alignments of the sample queries (Query seq.) and the target sequences were performed with T-COFFEE algorithm. Here, a representative local alignment obtained for *Ace2* and *Tmprss2* from the sequencing of the mixed samples from (**a**, **b**). In case of nucleotide non-determined, the symbol "N" is used. The level of the sequence conservation (Cons.) is indicated by asterisks and the color code scale. (**a**,**b**) H_2O is used as a negative control of transcript expression and *Gapdh* as a reporter gene. Ladder of 100 base pairs (bp, (**b**)).
